# Understanding the Gaps in Home Medication Safety: A Cross‐Sectional Study of Knowledge, Attitudes, and Behavioral Determinants

**DOI:** 10.1002/puh2.70395

**Published:** 2026-10-05

**Authors:** Asim Muhammed Alshanberi, Raghad Abdulmohsen Aljuhani, Husna Irfan Thalib, Adi Abdulrahman Alsharif, Zainab Jasim Alqurain, Abdulrahman Tawfeeg Abudawood, Safaa M. Alsanosi, Mable Pereira

**Affiliations:** ^1^ General Medicine Practice Program Batterjee Medical College Jeddah Kingdom of Saudi Arabia; ^2^ Department of Obstetrics and Gynecology King Faisal Specialist Hospital & Research Centre Riyadh Saudi Arabia; ^3^ Family and Community Medicine Department Faculty of Medicine in Rabigh King Abdulaziz University Jeddah Saudi Arabia; ^4^ Department of Pharmacology and Toxicology Faculty of Medicine Umm Al‐Qura University Makkah Saudi Arabia; ^5^ School of Medicine Lincoln American University Georgetown Guyana

**Keywords:** drug‐related side effects and adverse reactions, health knowledge, attitudes, practice, medication safety, Saudi Arabia, self medication

## Abstract

**Introduction:**

Home‐based medication use is common in the management of both acute and chronic health conditions. In Saudi Arabia, factors, such as education level, social background, and health literacy, may influence how medications are stored, used, and managed at home, thereby affecting medication safety. Previous studies have reported gaps in public knowledge, attitudes, and behaviors related to medication safety, including inappropriate use, storage, and adherence practices. Understanding these gaps within the community context is essential for improving safe medication use and reducing medication‐related harm.

**Aim:**

This study aims to assess these gaps among adults in Jeddah and suggest ways to improve safety and health outcomes.

**Methods:**

This cross‐sectional study was conducted in Jeddah, Saudi Arabia, between December 2023 and March 2024. A community‐based, self‐administered electronic questionnaire was used to assess adults’ knowledge, attitudes, and behaviors regarding home medication safety. The questionnaire consisted of closed‐ended items, including multiple‐choice questions. Convenience sampling was used, and ethical approval was obtained from the Institutional Review Board of Batterjee Medical College. Data analysis was conducted using IBM SPSS Version 25.

**Results:**

A total of 312 community‐dwelling adults from Jeddah, Saudi Arabia, participated (mean age 29.6 years), with the majority being female (69.2%), single (61.9%), and college‐educated (56.1%). Most participants reported taking two or fewer medications daily (82.1%) and once per day (75%). Mean scores were highest for medication safety knowledge (68.66%), followed by behavior (65.42%), whereas attitude scores were lowest (53.82%). Female participants had significantly higher knowledge scores than males (*p* < 0.0001), and higher medication counts were associated with lower knowledge and poorer behavior (*p* < 0.0001).

**Conclusion:**

The study highlights key gaps in how adults in Jeddah understand and manage medication at home. More awareness and educational efforts are needed to improve knowledge, encourage safer practices, and positively shift attitudes around medication use.

## Graphical abstract

**Background**: Home medication safety remains a public health concern. **Methods**: Cross‐sectional study of 312 adults in Jeddah assessed knowledge, attitudes, and behaviors related to home medication use. **Results**: Knowledge was highest (68.7%), behavior was moderate (65.4%), and attitudes were lowest (53.8%), indicating a critical gap. Female gender, fewer medications, and married status were associated with safer practices. **Conclusion**: Targeted health literacy, adherence support, and attitude‐focused interventions are essential to improve home medication safety and prevent medication‐related harm.

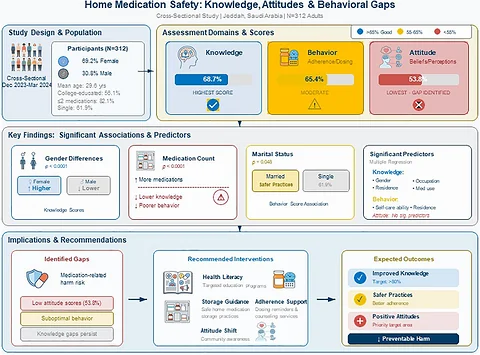

## 1 Introduction

Home‐based medication refers to the use of prescription and nonprescription medications taken by individuals in their homes without continuous direct supervision by healthcare professionals, including both prescribed therapies for chronic conditions and self‐medication practices. It is considered one of the mainstays of treatment in modern medical practice [1]. Its use has become increasingly common worldwide for managing both acute and chronic illnesses [2]. Although home‐based medication can effectively control many conditions and ease the financial burden on healthcare systems, it also carries significant risks. These include adverse drug events, harmful interactions, and incorrect dosing, all of which can have serious consequences, especially when driven by misuse or a lack of adequate knowledge [3].

Recent global literature has indeed revealed extensive gaps in knowledge, attitudes, and behaviors concerning the safe use of home‐based medications. For example, a study in Lilongwe, Malawi, found a high prevalence of self‐medication with antimicrobials, with 74% of respondents unable to distinguish antimicrobials from other medicines and 54% erroneously believing antimicrobials effectively treat common colds. Furthermore, 53% reported using antimicrobials for upper respiratory infections, indicative of poor knowledge and risky practices. The study also found that awareness of antimicrobial resistance (AMR) was low, and stocking antimicrobials at home significantly encouraged self‐medication, whereas awareness of AMR was associated with reduced self‐medication [4].

In the context of Saudi Arabia, cultural norms, social beliefs, educational background, and other contextual factors can significantly influence how medications are used at home. These variables may reduce medication effectiveness or, in some cases, contribute to harmful outcomes. Studies conducted within the Kingdom have documented specific patterns of home‐based medication use, revealing substantial gaps in public knowledge, attitudes, and behaviors regarding medication safety. For instance, a national survey found that more than half of participants lacked adequate knowledge about proper medication storage, whereas around 40% reported sharing prescription medications with family members [3, 4, 5]. These findings underscore the need for further investigation to better understand the practices and beliefs of the Saudi population and to identify strategies for improving safety in home care.

To address this gap, the present study aims to assess the levels of knowledge, attitudes, behaviors, and influencing factors related to home‐based medication safety among adults in Jeddah, Saudi Arabia. It also seeks to examine how incorrect knowledge and unsafe practices may expose individuals to medication‐related complications and to identify the sociodemographic, cultural, and behavioral factors that contribute to these risks. The results are intended to inform public health authorities, healthcare providers, and policymakers, enabling the development of targeted interventions to promote safer, more informed medication use in community settings.

Despite growing international attention to medication safety, community‐based evidence from Saudi Arabia remains limited. Existing research has largely focused on specific aspects such as medication storage or disposal or on hospital‐based populations, with minimal investigation of the knowledge, attitudes, and behaviors of community‐dwelling adults. Furthermore, the influence of behavioral determinants and sociodemographic factors on safe medication practices remains underexplored. Therefore, this study seeks to provide a comprehensive assessment of home‐based medication safety in a community setting, filling an important gap in local evidence and supporting strategies for improving patient safety in Saudi Arabia.

## 2 Materials and Methods

### 2.1 Study Design

A cross‐sectional analytical study was conducted in Jeddah, Saudi Arabia, between December 2023 and March 2024. The study employed a community‐based approach using a structured, self‐administered electronic questionnaire to assess knowledge, attitudes, and behaviors related to home‐based medication safety among adults. The reporting of this cross‐sectional study followed the Strengthening the Reporting of Observational Studies in Epidemiology (STROBE) statement (Table S1).

### 2.2 Study Population

The target population consisted of community‐dwelling adults aged 18 years and older living in Jeddah, Saudi Arabia, who reported current use of at least one medication taken at home. Medication use included prescription or nonprescription drugs used for acute or chronic conditions, reflecting real‐world home medication practices.

Inclusion criteria were adults aged ≥18 years, residing in the community (not hospitalized or institutionalized), who reported active use of one or more medications at home and who completed the questionnaire in full. Pregnant women were included because pregnancy commonly involves medication use (e.g., supplements and prescribed therapies) requiring safe home‐based management.

Exclusion criteria included individuals with severe cognitive impairment (e.g., Alzheimer's disease and advanced dementia) or communication limitations (severe hearing or speech impairment) that could compromise informed participation or accurate self‐reporting. Questionnaires with duplicate responses (identified by identical timestamps and responses) or substantial missing data were excluded from analysis.

Individuals who were acutely ill, hospitalized, or unable to provide informed consent were not eligible to participate, as the study aimed to assess medication safety practices among stable, home‐dwelling adults.

### 2.3 Questionnaire Tool

The survey questionnaire was adapted from the framework described in *Quality Indicators for Safe Medication Preparation and Administration* [6] and modified to assess home‐based medication safety among community‐dwelling adults. The final questionnaire consisted of four sections: sociodemographic characteristics, knowledge (16 items), attitude (8 items), and behavior (10 items) related to home medication safety.

Knowledge items were assessed using multiple‐choice and yes/no questions, whereas attitude items were measured using a five‐point Likert scale (ranging from strongly disagree to strongly agree). Behavior items primarily consisted of closed‐ended questions assessing routine medication storage, administration, and disposal practices.

Each domain was scored by summing the number of favorable responses. Because the three domains differed in the number of items and maximum possible scores, raw scores were converted into percentage scores (0–100) to allow comparability across knowledge, attitude, and behavior domains.

The original English questionnaire was translated into Arabic and back‐translated into English by independent bilingual language specialists to ensure semantic equivalence. Cultural and contextual adaptations were made to improve clarity and relevance for Saudi adults, including simplification of medical terminology, modification of examples to reflect locally available medications, and adjustment of wording to align with common home medication practices in Saudi Arabia.

A pilot study involving 10 participants was conducted to assess clarity, feasibility, and preliminary reliability. Internal consistency reliability was evaluated using Cronbach's alpha, which yielded values exceeding 0.6 across domains, indicating minimally acceptable reliability for exploratory research. Given the small pilot sample size, this reliability assessment was considered preliminary and is acknowledged as a study limitation.

To further support content validity, the questionnaire was reviewed by subject‐matter experts in pharmacology and community medicine, who assessed item relevance, clarity, and appropriateness for the study population. Feedback from these experts informed minor revisions to wording and item structure prior to final deployment. The full list of questionnaire items and response options is provided in Table S2.

### 2.4 Sample Size and Data Collection

The required sample size was calculated using the Raosoft sample size calculator [7], assuming a 95% confidence level, 5% margin of error, and an estimated population size of 20,000 adults. A population proportion of 25% was selected on the basis of previous regional studies reporting suboptimal medication safety knowledge and practices in community settings. To account for potential nonresponse, incomplete questionnaires, and exclusions during data cleaning, the calculated sample size was increased, resulting in a final target sample of 312 participants.

A convenience sampling approach was employed. Participants were recruited through an online, self‐administered questionnaire distributed via social media platforms, including WhatsApp, Telegram, and Twitter, which are commonly used by adults in Saudi Arabia. Recruitment was facilitated by four trained medical students, who shared the survey link within personal networks, general community groups, and publicly accessible social media forums targeting adult residents of Jeddah. Participation was limited to community‐dwelling adults aged 18 years or older who reported current use of at least one medication at home, regardless of whether the medication was for an acute or chronic condition.

Given the non‐probability sampling method, the study acknowledges the potential for selection bias and limited generalizability, as participants may not fully represent the broader adult population in Saudi Arabia.

Data were collected using Google Forms, with settings configured to restrict multiple submissions from the same account. Responses were screened for duplicate entries, incomplete questionnaires, and implausible patterns prior to analysis. Questionnaires with substantial missing data or duplicate responses were excluded. Cleaned data were securely stored and subsequently exported to Microsoft Excel for coding and preparation prior to statistical analysis.

### 2.5 Data Analysis

Statistical analysis was performed using SPSS version 25 (IBM Corp., Armonk, NY, USA). Descriptive statistics were used to summarize participants’ sociodemographic characteristics and medication‐related variables and are presented as frequencies and percentages.

The primary outcome variables were knowledge, attitude, and behavior scores related to home‐based medication safety. These variables were selected because they directly reflect the study objectives and are commonly used indicators in medication safety research. Scores for each domain were calculated by summing favorable responses within that domain. Because the three domains had different maximum possible scores (knowledge: 16 items; attitude: 8 items; and behavior: 10 items), all raw scores were converted into percentage scores (0–100) to enable comparability across domains and allow consistent application of inferential statistical tests.

Prior to inferential analyses, assumptions underlying parametric statistical testing were evaluated. The normality of percentage scores for knowledge, attitude, and behavior was assessed. Although the Kolmogorov–Smirnov and Shapiro–Wilk tests indicated statistically significant deviations from normality, these tests are known to be highly sensitive to sample size. Therefore, normality was further evaluated using graphical methods (histograms and Q–Q plots) and by examining skewness and kurtosis values, which indicated approximate normal distributions. Given the robustness of parametric statistical methods to modest violations of the normality assumption, parametric analyses were deemed appropriate and subsequently applied. To confirm the robustness of the results, corresponding nonparametric analyses were also conducted and produced comparable findings, indicating that the choice of parametric methods did not influence the study conclusion.

An independent samples *t*‐test was used to compare mean percentage scores for knowledge, attitude, and behavior between male and female participants. One‐way analysis of variance (ANOVA) was conducted to examine differences in mean percentage scores across categorical independent variables, including age group, marital status, educational level, monthly income, number of medications taken per day, and frequency of daily medication intake. These variables were selected on the basis of their theoretical relevance and prior evidence suggesting potential associations with medication safety practices.

To further explore factors associated with medication safety outcomes, multiple linear regression analyses were performed separately for knowledge, attitude, and behavior percentage scores as dependent variables. Independent variables entered into the models included gender, age group, marital status, education level, income category, number of medications taken per day, and frequency of medication intake. Regression assumptions, including linearity, the absence of multicollinearity, normality of residuals, and homoscedasticity, were evaluated and found to be acceptable.

All statistical tests were two‐tailed, and statistical significance was set at *p* < 0.05.

Boxplots were generated to visually illustrate the distribution and variability of knowledge, attitude, and behavior percentage scores, allowing comparison of score dispersion across the three medication safety domains.

### 2.6 Ethical Considerations

This study adhered to the ethical principles outlined in the Declaration of Helsinki. Ethical approval was obtained from the Institutional Review Board and Research Ethics Committee of Batterjee Medical College, Jeddah, Saudi Arabia (reference number RES‐2023‐0074). At the beginning of the questionnaire, participants were provided with the following consent statement.

## 3 Results

### 3.1 Sociodemographic Data

A total of 312 community‐dwelling adults from Jeddah, Saudi Arabia, were included in the analysis. The mean age of participants was 29.62 years, with the majority aged 18–24 years (59.3%). Most participants were female (69.2%), single (61.9%), and had a college‐level education (56.1%). The majority reported taking fewer than two medications per day (82.1%), and most reported taking medications once daily (75%) (Table 1).

**TABLE 1:** Sociodemographic and medication‐related characteristics of participants.

Demographic	Number (%)
Age (years)	
18–24	185 (59.3)
25–34	37 (11.9)
35–44	38 (12.2)
45–54	34 (10.9)
>55	18 (5.7)
Gender	
Male	96 (30.8)
Female	216 (69.2)
Marital status	
Single	193 (61.9)
Married	108 (34.6)
Widowed	3 (1.0)
Divorced	8 (2.6)
Level of education	
Illiterate	6 (1.9)
Eighth grade or below	5 (1.6)
High school	67 (21.5)
College	175 (56.1)
Postgraduate	59 (18.9)
Monthly personal income (SAR)	
<3000	175 (56.1)
3000–5000	38 (12.2)
5000–8000	43 (13.8)
>8000	56 (17.9)
Number of medications per day	
<2 types	256 (82.1)
3–5 types	35 (11.2)
6–9 types	12 (3.8)
>10 types	9 (2.9)
Number of times a day various medications taken	
Once	234 (75)
Twice	57 (18.3)
Three times or more	21 (6.7)

The scores for the different dimensions of medication safety are presented in Figure 1. The knowledge dimension had the highest average score of 68.66 (SD = 6.60), followed by behavior with an average score of 65.42 (SD = 8.19). The attitude dimension had the lowest average score of 53.82 (SD = 7.54).

**FIGURE 1:**
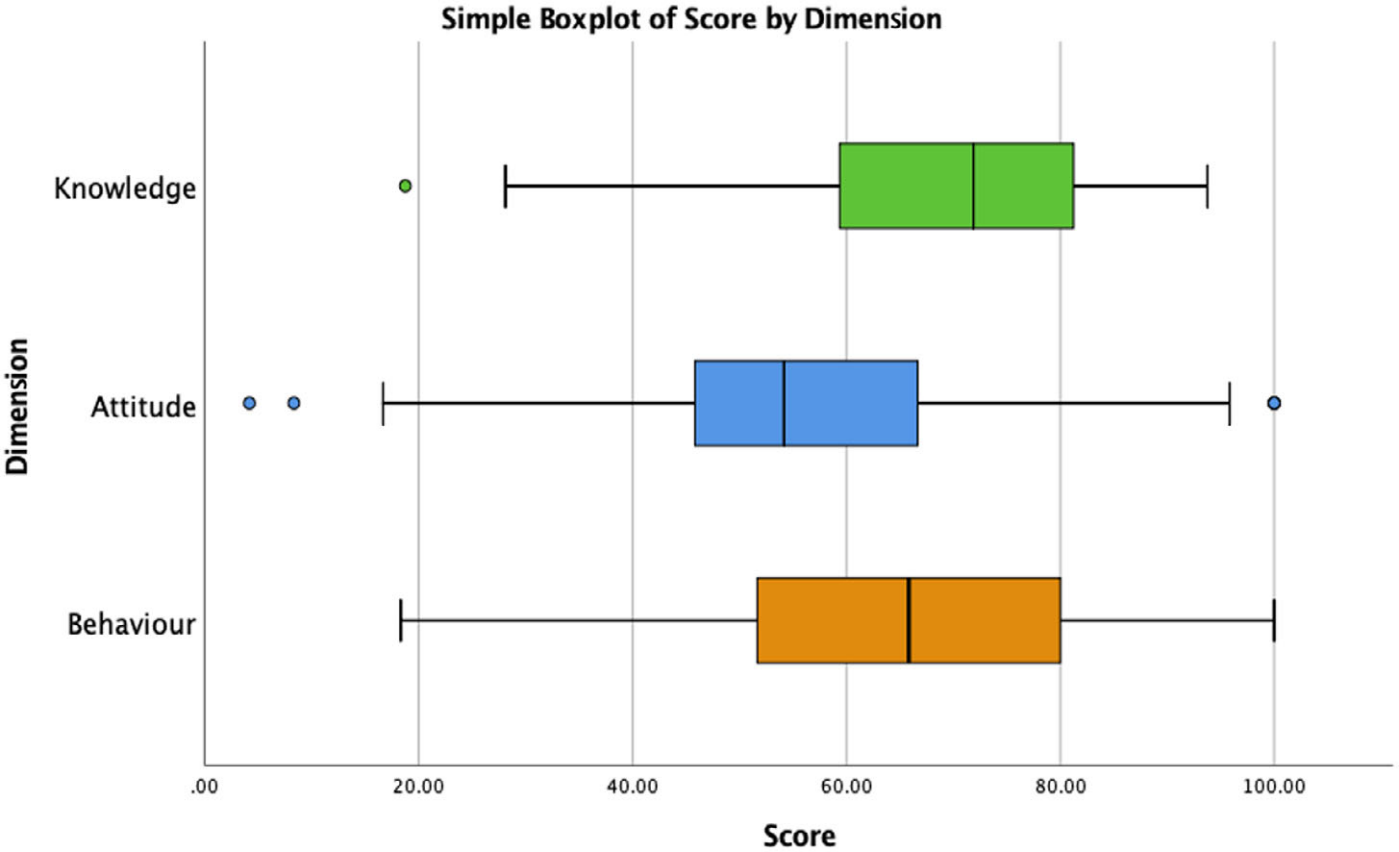
The dimension scores of medication safety.

### 3.2 Knowledge Towards Home‐Based Medications Safety

As shown in Table 2, knowledge scores related to home‐based medication safety did not differ significantly across age groups (*p* = 0.228), marital status (*p* = 0.362), educational level (*p* = 0.128), or monthly income (*p* = 0.168). Female participants demonstrated significantly higher knowledge scores than males (*p* < 0.0001). Knowledge scores were also significantly associated with the number of medications taken per day (*p* < 0.0001), with participants taking a higher number of medications generally exhibiting lower knowledge scores. In addition, frequency of medication intake per day was significantly associated with knowledge scores (*p* = 0.001). Post hoc analyses were not performed for nonsignificant ANOVA results.

**TABLE 2:** Comparing between demographic categories in the score of Knowledge.

		Knowledge	
Demographic	*N*	Mean (SD)	*p* value
Age (years)			0.228
18–24	185	70.7 (13.9)	
25–34	37	67.8 (17.4)	
35–44	38	65.5 (19)	
45–54	34	69.3 (15.6)	
>55	18	64.6 (19.8)	
Gender			<0.0001*
Male	96	65.2 (19)	
Female	216	71 (13.5)	
Marital status			0.362
Single	193	69.8 (15.4)	
Married	108	69 (15.7)	
Widowed	3	67.7 (17.2)	
Divorced	8	59.8 (18.8)	
Level of education			0.128
Illiterate	6	56.8 (7)	
Eighth grade or below	5	64.4 (16.3)	
High school	67	71.4 (13.2)	
College	175	69.8 (16.5)	
Postgraduate	59	66.2 (15.6)	
Monthly personal income (SAR)			0.168
<3000	175	70.4 (14.4)	
3000–5000	38	71.5 (14.6)	
5000–8000	43	66.4 (16.7)	
>8000	56	66.4 (18.6)	
Number of medications per day			<0.0001*
<2 types	256	71 (14.6)	
3–5 types	35	58.8 (18.2)	
6–9 types	12	69.5 (18.7)	
>10 types	9	59 (8.2)	
Number of times a day various medications taken			0.001^*^
Once	234	71.2 (13.9)	
Twice	57	63.5 (18.4)	
Three times or more	21	63.2 (20.4)	

*Note:* Independent samples *t*‐test was used for gender comparisons. One‐way ANOVA was used for comparisons involving age group, marital status, education level, income, number of medications per day, and frequency of medication intake.

* *p* < 0.05.

### 3.3 Attitude Towards Home‐Based Medications Safety

The mean attitude scores were compared across demographic and medication‐related variables (Table 3). No significant differences were observed between age groups (*p* = 0.999), genders (*p* = 0.934), or marital status groups (*p* = 0.163). Educational background (*p* = 0.515) and monthly personal income (*p* = 0.292) were also not significantly associated with attitude scores. Similarly, neither the number of medications taken per day (*p* = 0.192) nor the frequency of medication intake (*p* = 0.216) showed significant associations with attitude scores.

**TABLE 3:** Comparing between demographic categories in the score of Attitude.

		Attitude	
Demographic	*N*	Mean (SD)	*p* value
Age (years)			0.999
18–24	185	55.3 (16.9)	
25–34	37	55.2 (20)	
35–44	38	55.9 (17.9)	
45–54	34	55.8 (18.7)	
>55	18	56.3 (11.8)	
Gender			0.934
Male	96	53.5 (17.5)	
Female	216	56.4 (17.2)	
Marital status			0.163
Single	193	54.5 (17.4)	
Married	108	57.7 (16.9)	
Widowed	3	38.9 (26.8)	
Divorced	8	55.48 (17.3)	
Level of education			0.515
Illiterate	6	50 (21.1)	
Eighth grade or below	5	55 (20.3)	
High school	67	58.3 (17.5)	
College	175	55.3 (17.2)	
Postgraduate	59	53.3 (17.1)	
Monthly personal income (SAR)			0.292
<3000	175	55.3 (17.1)	
3000–5000	38	58.9 (13.5)	
5000–8000	43	57.3 (18.5)	
>8000	56	52.4 (19.2)	
Number of medications per day			0.192
<2 types	256	56 (16.7)	
3–5 types	35	51.9 (20.2)	
6–9 types	12	49.7 (18.4)	
>10 types	9	63 (20)	
Number of times a day various medications taken			0.216
Once	234	56.5 (17.3)	
Twice	57	52.7 (18.1)	
Three times or more	21	52 (14.2)	

A borderline negative association was observed between frequency of medication intake and attitude score (*B* = −3.32, *p* = 0.065); however, this regression coefficient should be interpreted cautiously as the type of regression was linear, unadjusted, and included only frequency of medication intake as a predictor (Table 3).

### 3.4 Behavior Towards Home‐Based Medications Safety

The behavior scores were compared across demographic and medication‐related variables (Table 4). No significant differences were observed for age (*p* = 0.248), gender (*p* = 0.336), educational background (*p* = 0.214), or monthly income (*p* = 0.168).

**TABLE 4:** Comparing between demographic categories in the score of behavior.

		Behavior	
Demographic	*N*	Mean (SD)	*p* value
Age (years)			0.248
18–24	185	64.7 (18.2)	
25–34	37	67.7 (18.2)	
35–44	38	67.8 (20.7)	
45–54	34	64.2 (18.6)	
>55	18	56.3 (25.1)	
Gender			0.336
Male	96	63 (20)	
Female	216	65.7 (18.6)	
Marital status			0.048^*^
Single	193	64.6 (18.8)	
Married	108	65.9 (18.7)	
Widowed	3	85.6 (8.4)	
Divorced	8	51.5 (25.7)	
Level of education			0.214
Illiterate	6	48.1 (7.6)	
Eighth grade or below	5	62.7 (15.1)	
High school	67	65.4 (17.5)	
College	175	65.9 (18.8)	
Postgraduate	59	63.1 (21.8)	
Monthly personal income (SAR)			0.168
<3000	175	64.8 (17.4)	
3000–5000	38	70.7 (18.9)	
5000–8000	43	61.6 (21.7)	
>8000	56	63.7 (21.2)	
Number of medications per day			<0.0001^*^
<2 types	256	67.1 (17.7)	
3–5 types	35	51.5 (21.2)	
6–9 types	12	65 (27.2)	
>10 types	9	54.3 (7)	
Number of times a day various medications taken			0.102
Once	234	66 (17.7)	
Twice	57	62.9 (21.3)	
Three times or more	21	57.5 (24.7)	

* *p* < 0.05.

Marital status was significantly associated with behavior scores (*p* = 0.048). However, the widowed group (*n* = 3) had an unusually high mean of 85.6 (SD = 8.4), whereas the divorced group (*n* = 8) had a low mean of 51.5 (SD = 25.7). These small sample sizes may produce unstable estimates, and results should be interpreted cautiously.

The number of medications per day was significantly associated with behavior scores (*p* < 0.0001). Linear regression showed a negative association (*B* = −4.91, *p* = 0.004), indicating that higher numbers of medications were associated with poorer behavior. The frequency of medication intake per day was not significantly associated with behavior scores (*p* = 0.102) (Table 4).

### 3.5 Multiple Linear Regression Analysis of Knowledge, Attitude, and Practice

Multiple linear regression analysis identified several factors significantly associated with knowledge scores. Gender was a significant predictor, with higher knowledge scores observed among one gender compared to the other (*B* = 4.44, *p* = 0.048). Residential status was also positively associated with knowledge (*B* = 1.78, *p* = 0.046). Occupation showed a significant positive association, indicating higher knowledge scores among certain occupational groups (*B* = 0.99, *p* = 0.009). In contrast, the number of medications taken per day (*B* = −3.04, *p* = 0.023) and the frequency of daily medication intake (*B* = −4.00, *p* = 0.009) were significantly associated with lower knowledge scores. Age, education level, marital status, self‐care ability, and monthly personal income did not show significant associations with knowledge (*p* > 0.05) (Table 5).

**TABLE 5:** Multiple linear regression coefficients for knowledge.

Predictor	*B*	SE	Beta	*t*	*p*	95% CI	
						Lower	Upper
(Constant)	59.862	7.959		7.521	<0.001	44.200	75.525
Gender	4.442	2.241	0.133	1.982	0.048^*^	0.031	8.853
Age (years)	0.765	1.033	0.065	0.740	0.460	−1.269	2.799
Education	0.180	1.150	0.009	0.157	0.876	−2.082	2.442
Marital status	1.771	1.774	0.074	0.998	0.319	−1.720	5.262
Residential status	1.782	0.890	0.126	2.001	0.046^*^	0.030	3.534
Occupation	0.990	0.376	0.157	2.632	0.009^**^	0.250	1.731
How well are you able to take care of yourself?	−2.615	1.492	−0.097	−1.752	0.081	−5.552	0.322
The number of medications you take a day?	−3.043	1.329	−0.133	−2.290	0.023^*^	−5.658	−0.428
The number of times a day do you take the various medications?	−4.001	1.523	−0.154	−2.627	0.009^**^	−6.999	−1.004
Monthly personal income?	−0.175	0.934	−0.013	−0.188	0.851	−2.014	1.663

*Note:* Dependent variable: Knowledge.

*p < 0.05.

**p < 0.01.

***p < 0.001.

In the multiple linear regression model for attitude, none of the examined predictors demonstrated a statistically significant association. Gender, age, education level, marital status, residential status, occupation, self‐care ability, number of medications taken per day, frequency of medication intake, and monthly personal income were not significantly associated with attitude scores (all *p* > 0.05). However, the frequency of daily medication intake showed a borderline association with attitude, though it did not reach statistical significance (*B* = −3.50, *p* = 0.053) (Table 6).

**TABLE 6:** Multiple linear regression coefficients for attitude.

Predictor	*B*	SE	Beta	*t*	*p*	95% CI	
						Lower	Upper
Constant	56.117	9.421		5.957	<0.001	37.578	74.656
Gender	3.463	2.653	0.092	1.305	0.193	−1.759	8.684
Age (years)	1.422	1.223	0.108	1.162	0.246	−0.985	3.829
Education	−1.048	1.361	−0.048	−0.770	0.442	−3.726	1.630
Marital status	0.585	2.100	0.022	0.279	0.781	−3.547	4.717
Residential status	−1.213	1.054	−0.076	−1.151	0.251	−3.287	0.861
Occupation	0.201	0.446	0.028	0.450	0.653	−0.676	1.077
How well are you able to take care of yourself?	1.608	1.767	0.053	0.910	0.363	−1.868	5.085
The number of medications you take a day?	−0.325	1.573	−0.013	−0.206	0.837	−3.420	2.770
The number of times a day do you take the various medications?	−3.497	1.803	−0.120	−1.940	0.053	−7.046	0.051
Monthly personal income?	−0.172	1.106	−0.012	−0.155	0.877	−2.348	2.004

*Note:* Dependent variable: Attitude.

*p < 0.05.

**p < 0.01.

***p < 0.001.

For practice scores, residential status emerged as a significant predictor, with individuals from certain residential settings demonstrating lower practice scores (*B* = −3.64, *p* = 0.004). Additionally, self‐care ability was positively associated with practice, indicating that participants who reported better ability to take care of themselves had higher practice scores (*B* = 4.75, *p* = 0.024). Other variables, including gender, age, education level, marital status, occupation, number of medications taken per day, frequency of medication intake, and monthly personal income, were not significantly associated with practice scores (*p* > 0.05) (Table 7).

**TABLE 7:** Multiple linear regression coefficients for behavior.

						95% CI	
Predictor	*B*	SE	Beta	*t*	*p*	Lower	Upper
Constant	52.374	11.166		4.690	<0.001	30.405	74.343
Gender	2.464	3.145	0.054	0.783	0.434	−3.727	8.655
Age (years)	2.078	1.449	0.130	1.434	0.153	−0.775	4.931
Education	−2.276	1.614	−0.086	−1.410	0.160	−5.452	0.900
Marital status	−0.605	2.488	−0.020	−0.243	0.808	−5.505	4.295
Residential status	−3.638	1.249	−0.191	−2.911	0.004^**^	−6.097	−1.179
Occupation	−0.599	0.529	−0.070	−1.133	0.258	−1.640	0.441
How well are you able to take care of yourself?	4.753	2.095	0.132	2.268	0.024^*^	0.627	8.879
The number of medications you take a day?	2.659	1.864	0.088	1.426	0.155	−1.012	6.330
The number of times a day do you take the various medications?	−2.897	2.137	−0.082	−1.355	0.177	−7.107	1.313
Monthly personal income?	−0.169	1.311	−0.010	−0.129	0.897	−2.752	2.414

*Note:* Dependent variable: Practice.

*p < 0.05.

**p < 0.01.

***p < 0.001.

## 4 Discussion

This study assessed knowledge, attitudes, and behaviors related to home‐based medication safety among community‐dwelling adults in Jeddah, Saudi Arabia. Overall, participants demonstrated the highest scores in knowledge, followed by behavior, whereas attitude scores were comparatively lower. This pattern suggests that although basic awareness of medication safety exists, it does not consistently translate into optimal attitudes or safe medication‐related behaviors.

Similar findings have been reported in previous studies conducted in China and other settings, where knowledge scores exceeded behavior and attitude scores, indicating a gap between awareness and practical implementation of medication safety principles [8, 9]. This discrepancy highlights the need for interventions that move beyond information provision and focus on behavioral change and attitude formation.

Gender differences were observed in this study, with males demonstrating higher knowledge scores than females, consistent with the results presented in the regression analysis. However, gender was also significantly associated with medication safety behaviors, underscoring the importance of considering sex‐specific factors when designing educational and behavioral interventions. These findings align with prior research suggesting that gender‐related differences in health literacy and medication management practices may influence medication safety outcomes [8]. Evidence from other populations suggests that this pattern may be explained less by gender per se than by underlying differences in health literacy and numeracy, which have been shown to mediate the relationship between gender and the capacity to manage a medication regimen accurately; this indicates that literacy‐ and numeracy‐focused education may be more effective than broadly targeted, gender‐based interventions alone [10, 11, 12, 13, 14, 15, 16, 17, 18, 19, 20, 21].

Medication burden emerged as a critical factor influencing medication safety. Participants who reported taking a higher number of medications per day had lower knowledge and poorer medication safety behaviors, indicating that polypharmacy may complicate patients’ understanding and management of medications. This finding is consistent with previous literature demonstrating that complex medication regimens are associated with increased risk of medication errors and reduced adherence [19]. These results emphasize the need for targeted counseling and simplified medication regimens, particularly for individuals managing multiple medications.

It is notable that most participants in this sample reported a relatively low medication burden, with 82.1% taking two or fewer medications daily, yet attitude and behavior scores remained suboptimal even within this lower‐burden group. This suggests that the challenges of home‐based medication management are not confined to individuals with high pill counts alone. Emerging evidence indicates that medication regimen complexity, which incorporates dosing frequency, number of daily administration times, dosage forms, and additional instructions, may be a stronger determinant of safe medication management than the number of medications alone. For example, in a large cohort of hospitalized patients with hypertension, a validated medication regimen complexity index, rather than the number of oral medications alone, independently predicted poor adherence and blood pressure control [20]. Applied to the present findings, this body of evidence implies that even a modest medication count can present meaningful management challenges when regimens involve variable dosing schedules, multiple formulations, or complex administration instructions. Future studies of home‐based medication safety would, therefore, benefit from incorporating validated regimen‐complexity measures alongside simple medication counts, rather than relying on medication count as a sole proxy for medication burden.

Marital status was also significantly associated with medication safety behaviors, with married participants exhibiting safer medication management practices compared to unmarried, divorced, or widowed individuals. This association may reflect the role of social support and shared responsibility in promoting safer health behaviors, as previously suggested in adherence and chronic disease management studies. This is consistent with a recent local study among hypertensive patients in Jeddah, which found that higher perceived social support and married status were both significantly associated with better medication adherence, reinforcing the relevance of family and social support structures to medication safety within this population [22].

In contrast, educational level was not significantly associated with knowledge, attitude, or behavior scores in this study. This finding suggests that formal education alone may not be sufficient to ensure safe medication practices and reinforces the importance of context‐specific health education that addresses practical medication use rather than general academic attainment.

Participants demonstrated relatively high awareness regarding the environmental and health risks associated with improper medication disposal, consistent with findings from studies conducted in Qatar and other regions [23, 24]. This level of awareness was higher than that reported in studies from Ethiopia and other low‐resource settings [25, 26, 27, 28], likely reflecting differences in public health messaging, access to healthcare information, and sociocultural context.

Taken together, these findings indicate that medication safety is influenced by a combination of individual knowledge, medication burden, and social factors rather than education alone. Addressing gaps in attitudes and behaviors, particularly among individuals managing multiple medications, should be a priority for community‐based medication safety interventions.

This study has several limitations. The use of convenience sampling and social media–based recruitment may limit representativeness and generalizability to the broader Saudi population, particularly older adults and individuals with limited digital access. Consistent with this concern, other recent Saudi web‐based surveys using similar convenience and social media–based recruitment have reported samples skewed towards younger, more educated, and predominantly female participants, a pattern also reflected in the present sample (mean age 29.6 years; 69.2% female; 56.1% college‐educated) [29]. This skew may limit the applicability of the findings to older adults, men, and individuals with lower educational attainment, groups who may face distinct or greater medication safety risks. In addition, as a cross‐sectional study, this design does not permit causal inference regarding the associations observed between sociodemographic factors and medication safety knowledge, attitudes, and behaviors; longitudinal or interventional studies are needed to establish directionality and to evaluate whether targeted interventions can improve these outcomes over time. In addition, reliance on self‐reported data introduces the potential for recall and social desirability bias. Self‐reported measures of medication‐related behavior are recognized to overestimate adherence and favorable practices relative to objective assessment methods, which suggests that the true prevalence of unsafe medication behaviors in this population may be underestimated rather than overestimated by our findings [30]. This study did not collect data on participants’ specific chronic disease diagnoses; therefore, the number of medications taken per day was used as a proxy measure of medication burden. As discussed above, this measure did not capture medication regimen complexity, such as dosing frequency, number of daily administration times, dosage forms, or additional instructions, which may influence medication safety independently of medication count [20]; future studies should incorporate validated regimen‐complexity indices alongside simple medication counts. The single‐timepoint survey format was also unable to capture change in participants’ knowledge, attitudes, or behaviors over time. Although the questionnaire demonstrated acceptable reliability for exploratory research, further validation using larger and more diverse samples is warranted. Finally, this study was conducted in a single city (Jeddah) over a defined period, and the findings may not generalize to other regions of Saudi Arabia that differ in healthcare access, cultural norms, or health education infrastructure; multicenter studies across diverse regions of the Kingdom are warranted to confirm the generalizability of these findings.

Despite these limitations, this study provides valuable insights into home‐based medication safety practices among adults in Saudi Arabia. The findings highlight specific gaps in attitudes and behaviors and identify medication burden and social factors as key determinants of safe medication use. These results support the development of targeted, community‐based education and counseling initiatives aimed at improving medication safety and reducing preventable medication‐related harm. Taken together with the broader literature, these findings suggest that future interventions should move beyond simply reducing the number of medications a patient takes and instead address the complexity of regimens, health literacy and numeracy, and the social support structures that surround home‐based medication management.

## 5 Conclusion

This study highlights a critical need to improve awareness and education on home‐based medication safety among adults in Jeddah, particularly among individuals using multiple medications. Significant associations with medication burden, gender, and marital status suggest that sociodemographic factors influence medication safety practices. These findings support the need for targeted, community‐based education and counseling initiatives, especially within primary care settings, to promote safer medication use at home.

## Author Contributions

Asim Muhammed Alshanberi, Raghad Abdulmohsen Aljuhani, Adi Abdulrahman Alsharif, Zainab Jasim Alqurain, Abdulrahman Tawfeeg Abudawood, Safaa M. Alsanosi, Husna Irfan Thalib, and Mable Pereira equally contributed to conceptualization, data collection, and writing of the manuscript. Asim Muhammed Alshanberi and Abdulrahman Tawfeeg Abudawood contributed to the data analysis, conceptualization, supervision, reviewing, and editing of the final manuscript.

## Funding

The authors have nothing to report.

## Conflicts of Interest

The authors declare no conflicts of interest.

## Supporting information




**Supporting File**: puh270395‐sup‐0001‐SuppMat.docx.

## Data Availability

Data will be available on request to authors due to privacy concern.
