# Supplementary material for: Understanding the Gaps in Home Medication Safety: A Cross‐Sectional Study of Knowledge, Attitudes, and Behavioral Determinants
Source: Public Health Chall. 2026 Oct 5;5(4):e70395. doi: 10.1002/puh2.70395 (PMC13638771; doi:10.1002/puh2.70395)
Supplement: Supplementary file 1 [file PUH2-5-e70395-s001.docx]

**Supplementary Material**

**Supplementary Table S1. STROBE Checklist for Cross-Sectional Studies**

| **STROBE Item** | **Recommendation** | **Reported in Manuscript (Section/Page)** |
| --- | --- | --- |
| **Title & Abstract** |  |  |
| 1(a) | Indicate the study’s design in the title or abstract | Title and Abstract (Cross-sectional study stated) |
| 1(b) | Provide an informative and balanced summary of what was done and found | Abstract (Introduction, Methods, Results, Conclusion) |
| **Introduction** |  |  |
| 2 | Explain the scientific background and rationale | Introduction |
| 3 | State specific objectives, including prespecified hypotheses | Introduction (Aim of the study) |
| **Methods** |  |  |
| 4 | Present key elements of study design early in the paper | Materials and Methods – Study Design |
| 5 | Describe the setting, locations, and relevant dates | Materials and Methods – Study Design |
| 6(a) | Give eligibility criteria and selection methods | Materials and Methods – Study Population |
| 6(b) | For matched studies, give matching criteria | Not applicable (cross-sectional, unmatched) |
| 7 | Clearly define outcomes, exposures, predictors, confounders | Materials and Methods – Questionnaire Tool |
| 8 | Describe data sources and measurement methods | Materials and Methods – Questionnaire Tool |
| 9 | Describe efforts to address potential bias | Materials and Methods – Sample Size and Data Collection |
| 10 | Explain how study size was arrived at | Materials and Methods – Sample Size and Data Collection |
| 11 | Explain how quantitative variables were handled | Materials and Methods – Data Analysis |
| 12(a) | Describe all statistical methods | Materials and Methods – Data Analysis |
| 12(b) | Describe methods for subgroup and interaction analyses | Materials and Methods – Data Analysis |
| 12(c) | Explain how missing data were addressed | Materials and Methods – Sample Size and Data Collection |
| 12(d) | Describe analytical methods accounting for sampling strategy | Materials and Methods – Data Analysis |
| 12(e) | Describe sensitivity analyses | Materials and Methods – Data Analysis |
| **Results** |  |  |
| 13(a) | Report numbers of individuals at each stage | Results – Sociodemographic Data |
| 13(b) | Give reasons for non-participation | Results / Data Collection description |
| 13(c) | Consider use of a flow diagram | Not included (not required) |
| 14(a) | Give characteristics of study participants | Results – Table 1 |
| 14(b) | Indicate number of participants with missing data | Results – Data screening described |
| 15 | Report numbers of outcome events or summary measures | Results – Figures and Tables |
| 16(a) | Give unadjusted and adjusted estimates with precision | Results – Tables 2–7 |
| 16(b) | Report category boundaries when variables categorized | Results – Tables |
| 16(c) | Translate estimates into meaningful terms | Results and Discussion |
| 17 | Report other analyses done | Results – Regression Analyses |
| **Discussion** |  |  |
| 18 | Summarize key results with reference to objectives | Discussion (Opening paragraphs) |
| 19 | Discuss limitations of the study | Discussion – Limitations paragraph |
| 20 | Provide interpretation considering objectives and evidence | Discussion |
| 21 | Discuss generalizability | Discussion – Limitations |
| **Other Information** |  |  |
| 22 | Give funding source and role of funders | Funding statement |
| 23 | Declare conflicts of interest | Conflict of Interest statement |
| 24 | Ethical approval and consent | Ethical Considerations |

**Supplementary Table S2. Questionnaire Items**

| **Section** | **Item** | **Response Options** |
| --- | --- | --- |
| **I. Demographics** | Gender | Male, Female |
|  | Age | 18–24, 25–34, 35–44, 45–54, 55–64, 65 or over |
|  | Education | Illiterate, 8th grade or below, High school, College, Post-graduate, Others |
|  | Marital status | Single, never married; Married; Widowed; Divorced |
|  | Residential status | Live with spouse; Live with children; Live with spouse and children; Live alone |
|  | Self-care ability | Completely self-care; Partly self-care; Completely unable to self-care |
|  | Number of medications per day | Two types and below; Three to five types; Six to nine types; Ten types and above |
|  | Medication frequency per day | Once; Twice; Three times or above |
|  | Occupation | Employees of institutions; Employees of state-owned enterprises; Employees of private enterprises; Medical, educational, or scientific workers; Self-employed; Freelancers; Retired; Others |
|  | Monthly personal income | Less than 3000 RMB; 3000–5000 RMB; 5000–7000 RMB; 8000 RMB and above |

| **Section** | **Item** | **Response Options** |
| --- | --- | --- |
| **II. Knowledge** | Awareness of prescription vs over-the-counter medications | Yes, No, Neutral |
|  | Medications have both therapeutic and toxic effects | Yes, No, Neutral |
|  | Folk medicine can affect effectiveness of medications | Yes, No, Neutral |
|  | More types of medications improve treatment effectiveness | Yes, No, Neutral |
|  | Medication use in older adults should consider liver and kidney function | Yes, No, Neutral |
|  | Dosage and type of medication should not always be the same for all patients | Yes, No, Neutral |
|  | More types and doses increase risk of adverse drug reactions | Yes, No, Neutral |
|  | Drugs should not be discontinued immediately without guidance | Yes, No, Neutral |
|  | Take medications exactly as prescribed | Yes, No, Neutral |
|  | Can stop medications without physician’s permission when feeling well | Yes, No, Neutral |
|  | Know how to judge medication quality | Yes, No, Neutral |
|  | Do not use medications that change color or become damp | Yes, No, Neutral |
|  | Taking expired medications can be harmful | Yes, No, Neutral |
|  | Know how to store medications correctly | Yes, No, Neutral |
|  | Know the purpose of the medication being taken | Yes, No, Neutral |
|  | Safe to start medications based on friend/relative advice | Yes, No, Neutral |

| **Section** | **Item** | **Response Options** |
| --- | --- | --- |
| **III. Attitude** | Use leftover prescription medications for similar symptoms | Strongly agree, Agree, Neutral, Disagree, Strongly disagree |
|  | Benefits of medications outweigh harms | Strongly agree, Agree, Neutral, Disagree, Strongly disagree |
|  | Concern about long-term medication harms | Strongly agree, Agree, Neutral, Disagree, Strongly disagree |
|  | Fear of dependency from long-term medication | Strongly agree, Agree, Neutral, Disagree, Strongly disagree |
|  | Dietary supplements have no side effects | Strongly agree, Agree, Neutral, Disagree, Strongly disagree |
|  | Manage medications based on experience or advice from friends | Strongly agree, Agree, Neutral, Disagree, Strongly disagree |
|  | Expensive or new medications are more effective than cheaper/older ones | Strongly agree, Agree, Neutral, Disagree, Strongly disagree |
|  | Herbal/natural remedies are more effective than conventional medications | Strongly agree, Agree, Neutral, Disagree, Strongly disagree |

| **Section** | **Item** | **Response Options** |
| --- | --- | --- |
| **IV. Behavior** | Buy medications without prescription | Never, Occasionally, Often, Always |
|  | Read medication instructions before use | Never, Occasionally, Often, Always |
|  | Ask pharmacist/physician for details about medications | Never, Occasionally, Often, Always |
|  | Suspect side effects when experiencing unusual symptoms | Never, Occasionally, Often, Always |
|  | Change dosage/frequency without doctor’s consent | Never, Occasionally, Often, Always |
|  | Change type of medication without doctor’s consent | Never, Occasionally, Often, Always |
|  | Have a dedicated medicine cabinet | Yes, No, Neutral |
|  | Store oral and topical medications separately | Yes, No, Neutral |
|  | Regularly check expiry dates and medication quality | Yes, No, Neutral |
|  | Discard expired or spoiled medications | Yes, No, Neutral |
